# Factors associated with dental fluorosis in three zones of Ecuador

**DOI:** 10.4317/jced.55124

**Published:** 2019-01-01

**Authors:** Ana del Carmen Armas-Vega, Farith-Damián González-Martínez, Mercedes-Silvana Rivera-Martínez, María-Fernanda Mayorga-Solórzano, Valeria-Elizabeth Banderas-Benítez, Osmani-Fabricio Guevara-Cabrera

**Affiliations:** 1 PhD. MSc. DDs. Oral Health Research Center–CISO. Teacher, Universidad UTE. Avenida Mariscal Sucre y Mariana de Jesús. ZIP-Code: 170902. Quito, Ecuador; 2 PhD. MSc. DDs. Director of the Public Health Group. University of Cartagena, Colombia. Department of Research, School of Dentistry. Universidad de Cartagena, Colombia. Campus Zaragocilla. Cra 30 # 39b-192. Cartagena, Colombia; 3 DDs. Clinica Dental Artist. Universidad Central del Ecuador, Avenida América y Universitaria, Quito, Ecuador; 4 DDs, Clinica Advance. Universidad Internacional del Ecuador, Ignacio Lecumberry OE6-223 y Nela Martínez. Quito, Ecuador; 5 MSc. Ing. Chief Evaluation Officer, Universidad UTE, Campus Occidental. Avenida Mariscal Sucre y Mariana de Jesús. Quito, Ecuador; 6 DDs, Clinica Dental Artist. Universidad Central del Ecuador, Avenida América y Universitaria, Quito, Ecuador

## Abstract

**Background:**

To determine the prevalence of dental fluorosis in 10-12 year-old school children, in three provinces of the inter-andean Region of Ecuador: Imbabura, Pichincha and Chimborazo, as well as the relationship between certain factors, considering that the latest studies go back to the year 2009.

**Material and Methods:**

A cross-sectional and observational study was proposed. A sample of 599 was calculated at 95% of confidence considering population projections for children between 10 and 12 old of three zones of Ecuador. However, 608 school children, who had the acceptance and informed consent of their parents to participate, completed a survey about factors associated with dental fluorosis. Once the survey was completed, the vestibular surfaces of the upper and lower anterior teeth of the infant were photographed, following standardized distance and light procedures. Three evaluators, trained in the detection of fluorosis using the Thylstrup and Fejerskov index, analyzed the photographs. The Stata 13.0 software was used for the statistical analysis, with a level of significance of 5% and with a confidence interval of 95%. To relate the risk factor of fluorosis, a multinomial logistic model was used.

**Results:**

The prevalence of dental fluorosis was of 89.96%, with a greater presence of grade 2 TF. A positive statistical relationship and statistical significance was detected between dental fluorosis and consumption of bottled beverages. Also the amount of toothpaste used and its ingestion during brushing (*p* = 0.000) were analyzed.

**Conclusions:**

The populations evaluated, that are related to the consumption of bottled beverages and involuntary toothpaste ingestion, and have a high prevalence of a mild level of fluorosis.

** Key words:**Fluorosis, dental, risk factors, epidemiology.

## Introduction

Dental fluorosis is considered an endemic pathology (1), which appears with mottled enamel. It is associated with the incorporation of fluoride during the process of tooth formation and maturation (2). Its presence is bilateral and symmetrical on the enamel (3), depending on the period of dental development in which contact occurred (4). It appears with lines in the form of horizontal striations, without a defined pattern (5), ranging from an opaque white (6) to the total loss of the enamel (7). Clinical variations are related to histological changes, cataloged with the TF index (8). Fluorides are transported through the bloodstream and deposited in calcified tissues (9).

Health organizations worldwide recommend the use of fluoridated salt in countries where water with fluoride is not very effective, considering the cost that this process represents (10). In addition, fluorides are found in table salt and, in a natural way, in different types of aliments (11); for example: in drinking water, juices, soft drinks, tea (12), toothpaste and mouthwashes (13), in which the fluoride concentration varies from 500 to 1500 mg/L (14). Different health agencies worldwide recommend the use of gels and varnishes, in a professional manner (15).

The presence of fluorosis in various regions of Ecuador has been reported, especially in central highland areas (16); it is related to the appearance of high levels of fluoride in the water supply network; however, different studies conducted in the same region report a normal fluoride concentration (17), suggesting that there are other factors that produce dental fluorosis. For this reason, one of the goals of this study is to determine the relationship of these factors with the prevalence and severity of dental fluorosis in schoolchildren, between 10-12 years of age, from ¨Pimampiro¨, Imbabura Province; ¨Colta¨, Chimborazo Province, and ¨Quito¨, Pichincha Province.

## Material and Methods

A cross-sectional observational study was proposed to determine the rate of dental fluorosis. A sample of 599 participants with a 95% confidence was calculated considering the population projections for children between 10 and 12 years old of three zones of Ecuador according to the data of the population census of 2010 (INEC, 2010). In addition the sample of the target group consisted of 608 school children, who live in the cantons of Pimampiro, (Imbabura); Colta, (Chimborazo) and Quito, (Pichincha) Participants in the study were chosen at random. An Ecuadorian research ethics committee approved the project.

Subjects´ conditions for the study were: to reside from their birth on in the cantons, absence of current or pre-existing systemic diseases, voluntary and free participation, verified through an informed consent, and have their upper and lower permanent incisors, clinically visible without presence of restorations, dental caries or dental braces.

Previous to the study, an upper and lower anterior teeth cleaning was performed. An intraoral photographic record was made, using a Nikon SLR digital SLR camera D5700, 100 mm macro lens and a circular flash, aperture of the iris in f / 25, exposure time in 1/125, ISO 200, white balance in flash, lens in manual mode and flash in TTL and the same position. The photographs were identified in order to preserve anonymity, and later analyzed by three researchers trained and standardized in detecting histological changes (18) in dental surfaces with Kappa values, likewise values higher than 6.5 points are considered appropriate for this type of tests and with expertise in detection of dental fluorosis, according to the Thylstrup and Fejerskov (TF) index. From the evaluation of each photograph, for each of the observers that obtained a unique value using the Index of concordance. A survey with 13 polycotomic questions, with categorical and numerical answers, was validated in terms of adequacy and reliability with cultural, psychosocial and ethnic conditions of the volunteers, similar to the population in which it was performed twice in previous studies. The results of each of the questions were compared among themselves through the test-retest reliability and concurrent validity method. The survey was addressed to the children’s parents and representatives in the study. The aim was to know the place of residence (rural/urban area), kind of water and milk consumption 

counting as an alternative the water obtained from a natural source, potable, on-board; and of milk sold in a liquid or powder market, maternal, acquired directly from the producer, kind of packaged beverages, age and frequency of consumption, dietary intake and salt consumption, age at which tooth brushing and the use of toothpaste began, and from when on brushing was performed by the children themselves. Finally, three water supply samples were collected from the evaluated provinces protocols, to be analyzed in terms of fluorine content in mg/L, following established protocols.

-Statistical analysis

The data of the fluorosis index and the analysis of the supply water network were collected in excel tables specifically designed for the study, to be analyzed using the STATA software version 13.0 with a level of significance of 5% and with a confidence interval of 95% considering that they were ordinal variables. A multinomial logistic model was established to assert fluorosis index in different categories.

## Results

The sample consisted of 608 school children; 56.6% (344) of the participants came from the urban area and 43.4% (264) from the rural zone; 52.1% (317) male and 47.9% (291) female.36.2% (220) of the school students live in Chimborazo province, 34.9% (212) in Pichincha and 28.9% (176) in Imbabura ( Table 1).

**Table 1:**
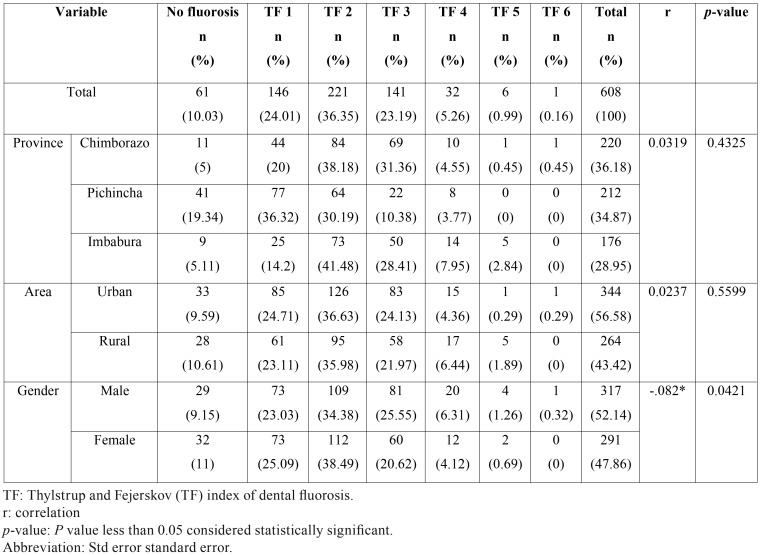
Specific descriptive results and composition of socio-demographic variables related to fluorosis index.

Prevalence of fluorosis reached 89.96%; with degree 2 TF being the most frequent in 36.35% of the cases, followed by degree 1 in 24.01% of them. In relation to the demographic variables, a negative correlation of 0.082 to 5% of dental fluorosis, was evident. No degrees 5 or 6 of dental fluorosis were found in Pichincha and Imbabura ( Table 1).

The data of the survey reports 38.8% consumption of tap water without boiling in the first 4 years of life 62.7% of entrants affirmed consumption of processed beverages after 3 years of age; juice appeared as the preferred beverage, with 44.2% of the cases, and consumed once a week in 51.8% of them. With relation to tooth brushing with adult paste after three years of age, 51.5% claim to have done it without the help of an adult. The use of adult toothpaste was reported by 54.9% of the participants ( Table 2).

**Table 2:**
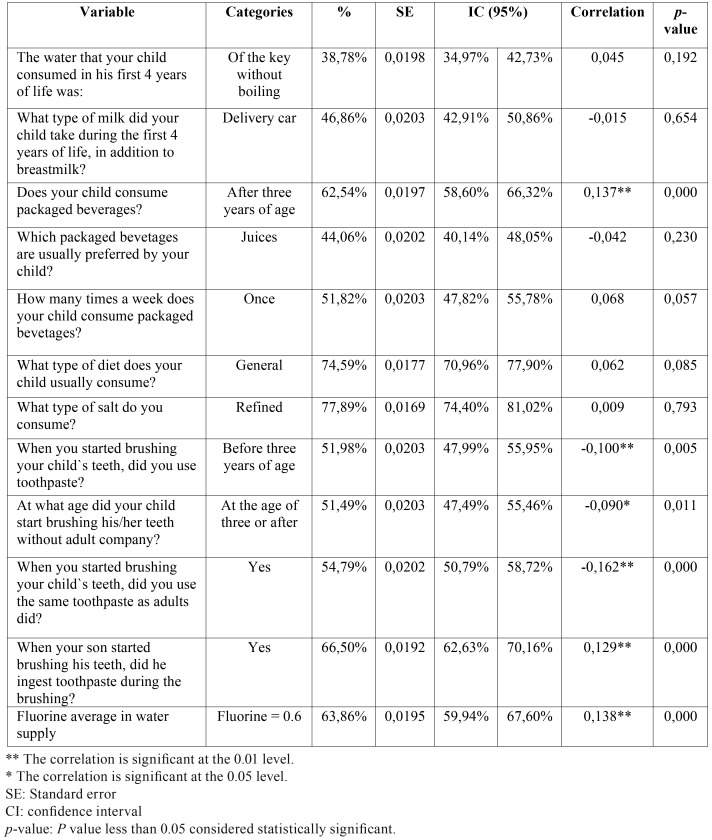
Specific descriptive results and composition of quality life variables.

The chemical analysis reported an average of fluorine of 0.752 mg / L in the examined waters. Clinical values demonstrated a positive relationship between dental fluorosis index with the consumption of processed beverages and ingestion of toothpaste during brushing. Evidence of a low negative relationship between the degree of fluorosis with the age of brushing with adult toothpaste during tooth brushing without help.

A multinomial logistic model was used to corroborate the results, relating the higher frequency of the elements evaluated and fluorosis degree, and an independent variables model to determine the influence.

A multinomial model was defined, considering: the fluorosis index (TF) as a dependent variable with seven categories from 0 to 6 for each, consumption of processed beverages, beginning of brushing with toothpaste, age of brushing without help, brushing with adult toothpaste, and the intake of toothpaste at the time of brushing. The average of fluorine in the water was considered as a continuous variable.

Considering the 1% of significance, in the statistical analysis, we observed a direct relationship between TF1, TF2, TF3 and TF4 indexes with an increased risk of presenting fluorosis was observed when brushing was done with adult toothpaste (OR:0.113, 0.111, 0.160, 0.042; *p*=0,000) and if there was an intake of it (OR: 25.392, 31.725, 23.159, 31.657; *p*=0,000). Odd ratios are much greater when children ingest toothpaste during the brushing ( Table 3).

**Table 3:**
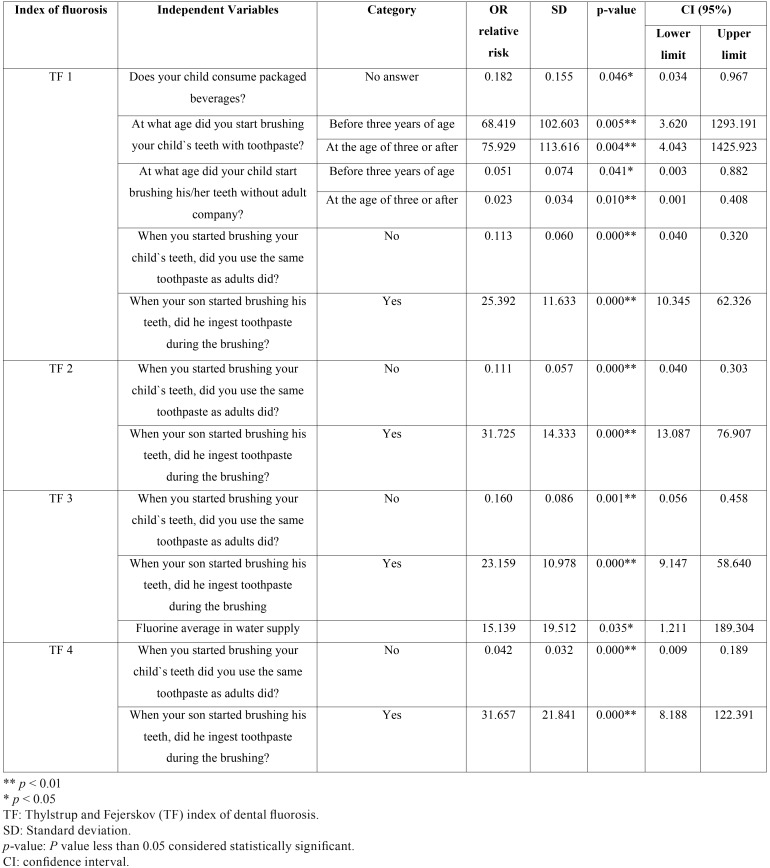
Increased risk of the multinomial model.

## Discussion

Study results reflect the presence of dental fluorosis in grade 2 TF as the most frequent, which agreed with previous studies carried out in other countries (19,20). In tap water, the percentage of fluorine refers values between 0.7 to 1.5 ppm, which are recommended as an international standard by the World Health Organization (WHO) (21). This is the opposite of previous results exposed in the same area (22) and in neighboring countries (23); the explanation could be the previous water treatments carried out by zonal governments, which had positive repercussions in the life quality of those who consumed it.

Relationship between the consumption of processed beverages, soft drinks and other sweetened beverages, with the presence of fluorosis, which could be explained by the fluoride content present in these beverages, reported in approximately 0.02 to 1.88 mg / L(24), which increases proportional to its frequency of consumption, obliging us as clinicians to alert the population about its use and consequences, especially by the presence of sugar in its composition that added to the hyperproteic and hypercaloric diet detected, are triggers of chronic non communicable diseases of the type diabetes or hypertension (25).

Regarding appropriate oral hygiene habits, one of the most important to maintain oral health is tooth brushing plus toothpaste; however, it could be the most determinant cause of the presence of fluorosis (26), as detected in the study, especially considering the fact that children started brushing their teeth with fluoride toothpaste for adults. 1450 mg / L of fluoride (27), with unmonitored and unregulated amount of toothpaste (11,23), is reflected in the correlation value of 0.162 detected, especially when considering that toothpastes marketed in the Ecuador have a percentage of fluorine that exceeds 1000 ppm which is beneficial from the preventive and protective point of view; however, it would be worth analyzing the amount used to confirm this association with the fluorosis detected.

Results indicate a hypercaloric and hyperproteic diet as the most frequent, which is related to the diet of the evaluated provinces (27), where fluorosis occurred in different degrees (28), with specific clinical manifestations (25). High levels of fluoride were not observed, which could be considered beneficial to the teeth (28); however, it is necessary to perform control processes of the composition of certain foods (24), as well as the involuntary intake of hygiene products (26), which could be related to the presence of the detected fluorosis.

This study was carried out considering the socio-demographic and ethnic conditions, which, conjugated with the standardization of the procedures performed, give us the possibility to guarantee results. The photographic record methodology for fluorosis degree has proved to be reliable and an ideal procedure, considering the visual fatigue of the clinical evaluator, due to the continuous and prolonged evaluation.

One of the strengths found in the study was the standardization among researchers responsible for detecting histological changes in dental surfaces, was evaluated both inter and intra observer; as well as the execution of this analysis in standardized photographs, avoiding visual exhaustion and misinterpretation of the evaluator.

It is extremely important to highlight the work that the health entities of Ecuador and the cantonal governments are doing, in order to achieve improvements in basic services. An example of this is the treatment of drinking water. However, it is necessary to develop studies with national specific strategies and unified methodologies that cover the majority of the population, including those areas especially considered as endemic of fluorosis in previous studies. The main goal of this work is to be able to count on reliable data of the current situation of the country in terms of its oral health and implement state policies.

Another point to consider is the presence of plaque, which although not evaluated in this study, was evidenced in an extreme way, indicating a poor execution of oral hygiene procedures that are in opposition to the use of toothpaste detected in the study. Hence, the importance of motivating and educating in oral hygiene practices at home and schools, as control strategies to prevent the massive destruction of future hard and soft tissues. New investigations regarding the presence of fluorosis and its relation to tooth brushing need to be carried out.

## Conclusions

In the evaluated sample, the prevalence of dental fluorosis is high and is present more frequently in mild degrees, with an association to the involuntary intake of toothpaste and / or consumption of processed beverages.
